# Dietary intake of yogurt and cheese in children at age 1 year and sleep duration at age 1 and 3 years: the Japan Environment and Children’s Study

**DOI:** 10.1186/s12887-022-03633-3

**Published:** 2022-11-01

**Authors:** Mariko Inoue, Kenta Matsumura, Narumi Sugimori, Kei Hamazaki, Akiko Tsuchida, Hidekuni Inadera, The Japan Environment and Children’s Study (JECS) Group

**Affiliations:** 1 Department of Public Health, Faculty of Medicine, University of Toyama, 2630 Sugitani, 930-0194 Toyama City, Toyama Japan; 2 Toyama Regional Center for JECS, University of Toyama, 2630 Sugitani, 930-8555 Toyama City, Toyama Japan; 3 Department of Public Health, Gunma University Graduate School of Medicine, Showa 3-39-22, 371-8511 Maebashi, Gunma Japan; 4 Graduate School of Medical Sciences Department of Occupational and Environmental Health, Nagoya City University, 1 Kawasumi, Mizuho-cho, Mizuho-ku, 467-8601 Nagoya, Aichi Japan

**Keywords:** Birth cohort, Sleep, Fermented foods

## Abstract

**Background:**

We previously reported that 1-year-old infants born to mothers who regularly consumed fermented food during pregnancy had a lower risk of sleep deprivation. However, it is not known if these positive effects are enhanced when infants themselves eat fermented foods or the long-term effects of such consumption. In this study, we examined the association between the frequency of fermented food intake during the child’s weaning period and sleep deprivation at age 1 and 3 years.

**Methods:**

This birth cohort study used data from a nationwide, government-funded study called the Japan Environment and Children’s Study (JECS), covering 65,210 mother-child pairs. We examined the association between infants’ consumption of fermented foods at 1 year of age and sleep deprivation at 1 and 3 years of age.

**Results:**

There was no association between yogurt or cheese intake and sleep duration at age 1; at age 3, there was no group difference, although a trend test showed that yogurt intake at age 1 was significantly associated with sleep duration at age 3. There was also no association between the frequency of cheese intake and inadequate sleep duration at age 3.

**Conclusion:**

Frequency of children’s yogurt and cheese intake at age 1 was not associated with sleep duration at age 1 or 3. However, a trend test showed a significant association between the frequency of yogurt intake at age 1 and sleep duration at age 3.

## Background

A sufficient amount of good sleep is necessary for a healthy life. Lack of sleep and irregular sleep patterns have been reported to increase the risk of physical illnesses [1], such as hypertension [2] and diabetes [3], and mental illnesses, such as depression [4, 5] and self-harm [6]. This is true not only for adults but also children, whose sleep duration varies from the neonatal period to infancy [7]. Sleep deprivation in infancy has been found to be associated with obesity [8], poor academic and spatial skills [9], hyperactivity [10], problematic behavior [11], and hyperactivity at age 6 years [12], and it has a negative impact on physical and psychological development. It is therefore important to investigate the causes and effects of sleep deprivation in infancy.

Probiotic-containing foods and fermented foods are gaining attention due to their positive effects on the gut microbiota [13, 14], and a good gut microbiota has a positive effect on sleep [15, 16]. Studies on probiotic-containing foods and/or the gut microbiota include a small study of 8 people in which consumption of yogurt-containing probiotics improved gut bacteria after antibiotic treatment [17]. In a study of 66 elderly people, a probiotic group treated daily for 6 months with a fermented flavored oat drink containing 109 cfu/mL *Bifidobacterium longum* 46 (DSM 14,583) and *B. longum* 2 C (DSM 14,579) also showed higher and more diverse levels of bifidobacteria in their stool [18]. Interestingly, in a study of the gut microbiota and sleep in 37 healthy elderly people, a higher proportion of *Verrucomicrobia* and *Lentisphaerae* in their stool was associated with better sleep quality and better Stroop performance [19]. A study of 9 men determined that a sleep-deprived group, which slept about 4 h, had lower total amounts of acetate, propionate, and fecal short-chain fatty acids in their stool, suggesting the importance of sleep duration and the composition of the gut microbiota [20]. Furthermore, a study of 40 men also reported that diversity of the microbiome in the gut microbiota was positively correlated with sleep efficiency and total sleep time and was negatively correlated with sleep fragmentation [21]. However, as yet, there is a lack of research involving children, particularly infants, a lack of large-scale studies, and a lack of research focusing directly on sleep from the perspective of dietary content.

The Japan Environment and Children’s Study (JECS), known as *Ecochil-Chosa* in Japan, is a nationwide birth cohort study investigating the environmental factors possibly affecting children’s health and development. A total of 104,059 pregnancies have been registered, and data from self-administered questionnaires and medical record transcriptions have yielded a wide array of research findings [22]. The JECS previously examined the association between the frequency of the maternal consumption of fermented foods during pregnancy and the infant’s sleep duration at 1 year of age in 72,624 mother-infant pairs [23]. Infants whose mothers consumed miso soup more often during pregnancy were found to sleep longer. Similar results were obtained for cheese and sleep at age 3 [24]. These results suggest that a high intake of fermented foods during pregnancy may have a positive effect on the child’s sleep. However, it is also conceivable that the child’s diet may have a greater impact on their development than the mother’s diet during pregnancy.

In this study, we focused on yogurt and cheese, which are often consumed in Japan as probiotic-containing fermented foods, and examined the association between frequency of intake at age 1 and sleep duration at ages 1 and 3.

## Methods

### JECS population

**Fig. 1:**
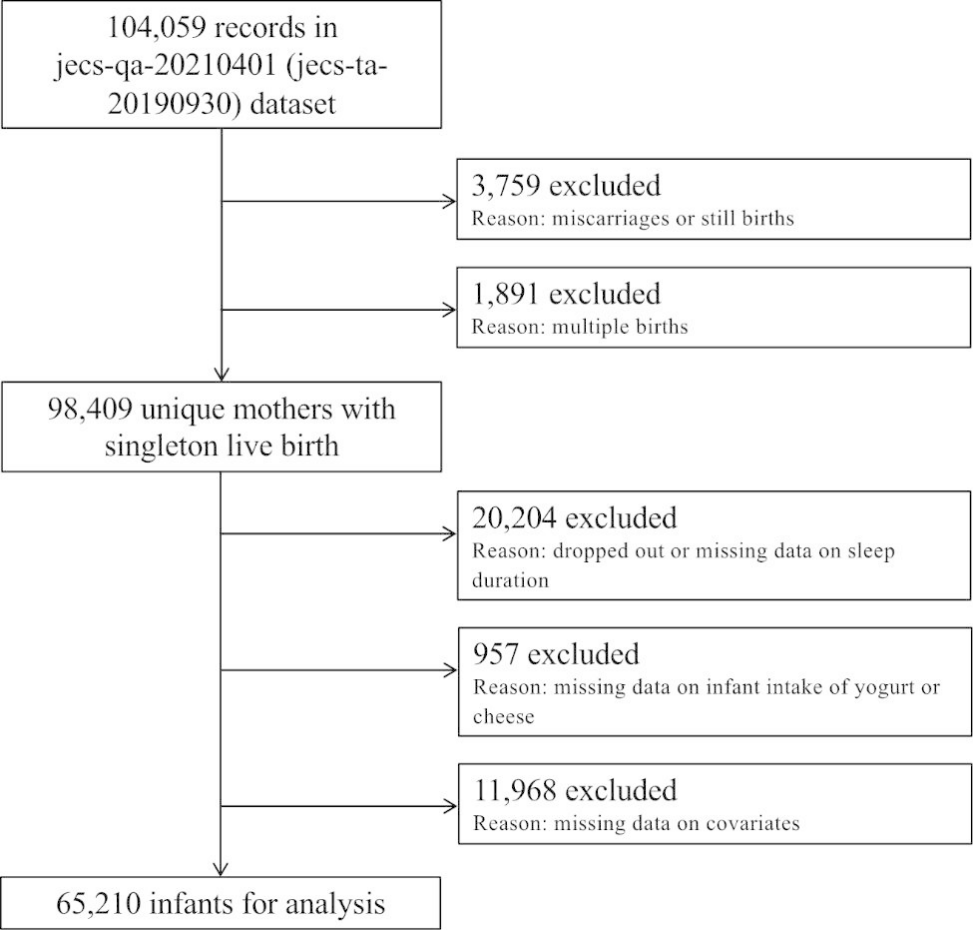
Flow diagram of the recruitment and exclusion process for participants

The JECS protocol has been described in detail elsewhere [25, 26]. Briefly, the aim of the JECS, a nationwide government-funded birth cohort study, is to determine the impact of certain environmental factors on child health and development. The JECS participants included women in the first trimester of pregnancy, belonging to 15 regions of Japan, who were enrolled between January 2011 and March 2014 [25, 26]. The eligibility criteria for participants (expectant mothers) were as follows: (1) resident of a Study Area at the time of recruitment and expected to reside continually in Japan for the foreseeable future; (2) expected delivery date between August 1, 2011, and mid-2014; and (3) able to participate in the study without difficulty (i.e., able to understand Japanese and complete the self-administered questionnaire). The excluded participants were expectant mothers residing outside the Study Area, even if they visited co-operating health care providers within a Study Area. The present study analyzed the jecs-qa-20210401 (jecs-ta-20190930) dataset, released in April 2021. The full dataset comprises 104,059 records obtained from a questionnaire-based survey of the participants. We excluded 3,759 and 1,891 records because of miscarriages/still births and multiple births, respectively (Fig. 1). Additionally, we excluded 20,204 participants with missing data on sleep duration and 957 participants with missing data on yogurt or cheese consumption.

### Exposure

To assess the frequency of probiotic intake, the following questions were included in a self-administered questionnaire that mothers completed 1 year after delivery: “How many times a week does your child have yogurt?” and “How many times a week does your child have cheese?” The response options were < 1 time/week, 1–2 days/week, 3–4 days/week, 5–6 days/week, 1 time/day, 2 times/day, and ≥ 3 times/day. Because of variation in the number of responses in the frequency categories for yogurt and cheese, categorization was performed by food. Specifically, we categorized participant responses for the consumption of yogurt by infants as < 1 time/week, 1–2 days/week, 3–6 days/week, or ≥ 7 times/week and for cheese as < 1 time/week, 1–2 times/week, or ≥ 3 times/week [27].

### Outcome categories

To measure infant sleep duration at 1 and 3 years after delivery, parents were asked in the questionnaire to indicate when their infant slept on the previous day. Parents marked the times when their infant was asleep by drawing lines through boxes indicating 30-min intervals from 12:00 am to 12:00 am the next day [23]. Infants were categorized according to tertile or quartile of fermented food intake to estimate their risk of sleep deprivation. The National Sleep Foundation in the United States recommends 11–14 h of sleep in a 24-h period for 1-year-olds and 10–13 h for 3-year-olds. Therefore, the lower limit of appropriate sleep duration was set at less than 11 h for 1-year-olds and 10 h for 3-year-olds [7].

### Confounders in multiple logistic regression analysis

The following confounding factors were used in multiple logistic regression: maternal age; body mass index (kg/m^2^) 1 month after delivery (< 18.5, 18.5–25, or ≥ 25); number of previous deliveries (nulliparous or multiparous); educational background (junior high school or high school, technical junior college, technical/vocational college or associate degree, or Bachelor’s degree or postgraduate degree); annual household income (< 4 million JPY, 4–6 million JPY, or ≥ 6 million JPY); marriage status 6 months after delivery (married, divorced, widowed, or other); alcohol status 1 month after delivery (never, ex-drinker, 1–3 times/month, 1–3 times/week, 4–6 times/week, or every day); smoking status 1 month after delivery (never, quit before learning of pregnancy, quit after learning of pregnancy, currently smoking (≤ 10 cigarettes or ≥ 10 cigarettes); employment status 1 year after delivery (employed or unemployed); cesarean section (no or yes); gestational age at birth (weeks); birth weight (g); infant sex (male or female); major congenital anomaly (no or yes); birth season (spring, summer, fall, or winter), night crying at 1 year of age (no or yes); and attending nursery school at 1 year of age (no or yes). These variables were categorized according to standard medical practice or common practice in Japan [28, 29] and as shown in Tables 1 and 2, and 3.

### Statistical analysis

Unless stated otherwise, data are expressed as mean ± standard deviation or median. Univariate and multivariate logistic analyses were applied to estimate the incidence of inadequate sleep duration (< 11 h at 1 year of age and < 10 h at 3 years of age). Four logistic analyses were conducted to determine the association between the frequency of yogurt intake at age 1 and sleep at age 1, frequency of yogurt intake at age 1 and sleep at age 3, frequency of cheese intake at age 1 and sleep at age 1, and frequency of cheese intake at age 1 and sleep at age 3[24]. We calculated both unadjusted and adjusted odds ratios (ORs) with 95% confidence intervals (95% CIs).

ORs and 95% CIs were calculated using logistic regression analysis with yogurt as the lowest quartile criterion and cheese as the lowest tertile criterion. Adjusted ORs were calculated using the covariates described in the previous section, and crude ORs were calculated without these covariates. The JECS prohibits the sharing of the ORs of covariates, regardless of whether they are crude or adjusted. Trend tests were conducted with yogurt and cheese intake respectively assessed using quartile and tertile distributions as continuous variables. All statistical analyses were performed by using SAS version 9.4 (SAS Institute Inc., Cary, NC).

## Results

Demographic and obstetric characteristics of participants (n = 65,210) are shown in Tables 1 and 2. The group with higher yogurt consumption was more likely to be primiparous, to have a higher household income, and not to attend nursery school. On the other hand, the group that consumed more cheese tended to be primiparous and more educated and to have a higher household income; in addition, the mothers tended to be unemployed and the infants tended to not attend nursery school.

**Table 1:** Participant characteristics by frequency of infant consumption of yogurt at 1 year of age

		Yogurt consumption at 1 year of age, times/week												
		< 1			1–2					3–6			≥ 7	
		(n = 13,443)			(n = 20,612)					(n = 19,811)			(n = 11,344)	
		n	(%)		n	(%)				n	(%)		n	(%)
**Mother’s age at childbirth**														
	Mean ± SD, y	31.6 ± 4.81			31.3 ± 4.83					31.6 ± 4.77			32.1 ± 4.75	
**Pre-pregnancy BMI, kg/m** ^**2**^														
	< 18.5	701	(5.2)		905	(4.4)				1,002	(5.1)		662	(5.8)
	18.5 to < 25	10,770	(80.1)		16,409	(79.6)				15,968	(80.6)		9,053	(79.8)
	≥ 25	1,972	(14.7)		3,298	(16.0)				2,841	(14.3)		1,629	(14.4)
**Parity**														
	Primiparous	4,720	(35.1)		7,256	(35.2)				8,989	(45.4)		6,374	(56.2)
	Multiparous	8,723	(64.9)		13,356	(64.8)				10,822	(54.6)		4,970	(43.8)
**Highest education level, y**														
	≤ 12	4,392	(32.7)		7,283	(35.3)				6,036	(30.5)		3,202	(28.2)
	12 to < 16	5,641	(42.0)		8,826	(42.8)				8,824	(44.5)		5,092	(44.9)
	≥ 16	3,410	(25.4)		4,503	(21.9)				4,951	(25.0)		3,050	(26.9)
**Annual household income, JPY**														
	< 4 million	5,416	(40.3)		8,399	(40.8)				7,189	(36.3)		3,794	(33.4)
	4 to < 6 million	4,533	(33.7)		6,981	(33.9)				6,788	(34.3)		3,792	(33.4)
	≥ 6 million	3,494	(26.0)		5,232	(25.4)				5,834	(29.5)		3,758	(33.1)
**Marital status**														
	Married	13,262	(98.7)		20,317	(98.6)				19,580	(98.8)		11,195	(98.7)
	Divorced or widowed	181	(1.4)		295	(1.4)				231	(1.2)		149	(1.3)
**Alcohol intake**														
	Never	12,323	(91.7)		18,761	(91.0)				18,251	(92.1)		10,550	(93.0)
	Former	563	(4.2)		944	(4.6)				870	(4.4)		467	(4.1)
	Current	557	(4.1)		907	(4.4)				690	(3.5)		327	(2.9)
**Smoking history**														
	Never	8,241	(61.3)		12,195	(59.2)				12,324	(62.2)		7,327	(64.6)
	Quit	4,764	(35.4)		7,645	(37.1)				6,930	(35.0)		3,767	(33.2)
	Current	438	(3.3)		772	(3.8)				557	(2.8)		250	(2.2)
**Employed**														
	No	7,272	(54.1)		10,210	(49.5)				10,164	(51.3)		6,179	(54.5)
	Yes	6,171	(45.9)		10,402	(50.5)				9,647	(48.7)		5,165	(45.5)
**Cesarean section**														
	No	10,928	(81.3)		16,989	(82.4)				16,170	(81.6)		9,129	(80.5)
	Yes	2,515	(18.7)		3,623	(17.6)				3,641	(18.4)		2,215	(19.5)
**Gestational weeks**														
	Mean ± SD, weeks	39.3 ± 1.55			39.3 ± 1.44					39.3 ± 1.46			39.3 ± 1.47	
**Birth weight**														
	Mean ± SD, kg	3035 ± 413.5			3036 ± 402.9					3028 ± 402.9			3016 ± 403.5	
**Infant sex**														
	Male	6,987	(52.0)		10,324	(50.1)				10,048	(50.7)		5,922	(52.2)
	Female	6,456	(48.0)		10,288	(49.9)				9,763	(49.3)		5,422	(47.8)
**Major congenital anomaly**														
	No	13,150	(97.8)		20,176	(97.9)				19,364	(97.7)		11,074	(97.6)
	Yes	293	(2.2)		436	(2.1)				447	(2.3)		270	(2.4)
**Birth season**														
	Spring (months 3–5)	3,000	(22.3)		4,782	(23.2)				4,756	(24.0)		2,731	(24.1)
	Summer (months 6–8)	3,381	(25.2)		5,551	(26.9)				5,488	(27.7)		3,228	(28.5)
	Fall (months 9–11)	3,856	(28.7)		5,664	(27.5)				5,341	(27.0)		2,969	(26.2)
	Winter (months 12–2)	3,206	(23.9)		4,615	(22.4)				4,226	(21.3)		2,416	(21.3)
**Night crying at 1 year of age**														
	No	6,827	(50.8)		10,679	(51.8)				10,150	(51.2)		5,883	(51.9)
	Yes	6,616	(49.2)		9,933	(48.2)				9,661	(48.8)		5,461	(48.1)
**Nursery attendance at 1 year of age**														
	No	9,874	(73.5)		14,157	(68.7)				14,727	(74.3)		9,094	(80.2)
	Yes	3,569	(26.6)		6,455	(31.3)				5,084	(25.7)		2,250	(19.8)

BMI, body mass index

JPY, Japanese Yen

**Table 2:** Participant characteristics by frequency of infant consumption of cheese at 1 year of age

		Cheese consumption at 1 year of age, times/week							
		< 1			1–2			≥ 3	
		(n = 30,614)			(n = 26,178)			(n = 8,418)	
		n	(%)		n	(%)		n	(%)
**Mother’s age at childbirth**									
	Mean ± SD, y	31.6 ± 4.79			31.5 ± 4.82			32.0 ± 4.75	
**Pre-pregnancy BMI, kg/m** ^**2**^									
	< 18.5	1,592	(5.2)		1,218	(4.7)		460	(5.5)
	18.5 to < 25	24,373	(79.6)		21,050	(80.4)		6,777	(80.5)
	≥ 25	4,649	(15.2)		3,910	(14.9)		1,181	(14.0)
**Parity**									
	Primiparous	12,277	(40.1)		10,933	(41.8)		4,129	(49.1)
	Multiparous	18,337	(59.9)		15,245	(58.2)		4,289	(51.0)
**Highest education level, y**									
	≤ 12	9,974	(32.6)		8,650	(33.0)		2,289	(27.2)
	12 to < 16	13,248	(43.3)		11,393	(43.5)		3,742	(44.5)
	≥ 16	7,392	(24.2)		6,135	(23.4)		2,387	(28.4)
**Annual household income, JPY**									
	< 4 million	12,044	(39.3)		9,833	(37.6)		2,921	(34.7)
	4 to < 6 million	10,266	(33.5)		8,929	(34.1)		2,899	(34.4)
	≥ 6 million	8,304	(27.1)		7,416	(28.3)		2,598	(30.9)
**Marital status**									
	Married	30,229	(98.7)		25,809	(98.6)		8,316	(98.8)
	Divorced or widowed	385	(1.3)		369	(1.4)		102	(1.2)
**Alcohol intake**									
	Never	28,337	(92.6)		23,831	(91.0)		7,717	(91.7)
	Former	1,238	(4.0)		1,198	(4.6)		408	(4.9)
	Current	1,039	(3.4)		1,149	(4.4)		293	(3.5)
**Smoking history**									
	Never	19,205	(62.7)		15,622	(59.7)		5,260	(62.5)
	Quit	10,489	(34.3)		9,645	(36.8)		2,972	(35.3)
	Current	920	(3.0)		911	(3.5)		186	(2.2)
**Employed**									
	No	15,911	(52.0)		13,175	(50.3)		4,739	(56.3)
	Yes	14,703	(48.0)		13,003	(49.7)		3,679	(43.7)
**Cesarean section**									
	No	24,943	(81.5)		21,365	(81.6)		6,908	(82.1)
	Yes	5,671	(18.5)		4,813	(18.4)		1,510	(17.9)
**Gestational weeks**									
	Mean ± SD, weeks	39.2 ± 1.56			39.3 ± 1.41			39.3 ± 1.36	
**Birth weight**									
	Mean ± SD, kg	3029 ± 415.7			3032 ± 399.1			3028 ± 385.6	
**Infant sex**									
	Male	15,646	(51.1)		13,319	(50.9)		4,316	(51.3)
	Female	14,968	(48.9)		12,859	(49.1)		4,102	(48.7)
**Major congenital anomaly**									
	No	29,913	(97.7)		25,625	(97.9)		8,226	(97.7)
	Yes	701	(2.3)		553	(2.1)		192	(2.3)
**Birth season**									
	Spring (months 3–5)	6,927	(22.6)		6,332	(24.2)		2,010	(23.9)
	Summer (months 6–8)	8,036	(26.3)		7,264	(27.8)		2,348	(27.9)
	Fall (months 9–11)	8,655	(28.3)		6,959	(26.6)		2,216	(26.3)
	Winter (months 12–2)	6,996	(22.9)		5,623	(21.5)		1,844	(21.9)
**Night crying at 1 year of age**									
	No	15,758	(51.5)		13,492	(51.5)		4,289	(51.0)
	Yes	14,856	(48.5)		12,686	(48.5)		4,129	(49.1)
**Nursery attendance at 1 year of age**									
	No	22,601	(73.8)		18,634	(71.2)		6,617	(78.6)
	Yes	8,013	(26.2)		7,544	(28.8)		1,801	(21.4)

BMI, body mass index

JPY, Japanese Yen

The unadjusted and adjusted ORs (95% CIs) for the relationship of inadequate sleep duration with yogurt and cheese consumption at 1 and 3 years of age are shown in Table 3. In terms of frequency of yogurt intake at age 1 and sleep duration at age 3, the incidence of children with sleep deprivation decreased when yogurt was consumed ≥ 7 times per week in the crude model. The adjusted model showed no differences between groups, although a trend test showed significant differences. In all other conditions, there were no significant differences in both the crude and adjusted models.

**Table 3:** ORs (95% CIs) for inadequate sleep duration at 1 and 3 years of age according to frequency of infant consumption of yogurt and cheese at 1 year of age (n = 65,210)

			Cases,	Controls,		Crude model^a^		Adjusted model^a, b^	***p***-value for trend^c^
			**n**	**n**		**Odds ratios (95% CI)**		**Odds ratios (95% CI)**	
**Sleep deprivation at 1 year of age (cross-sectional design)**									
	**Yogurt, times/week**								
		< 1	1,261 /	12,182			Reference	Reference	0.793
		1–2	1,988 /	18,624			1.031 (0.938–1.133)	1.000 (0.909–1.100)	
		3–6	1,766 /	18,045			0.945 (0.858–1.041)	0.939 (0.852–1.035)	
		≥ 7	1,022 /	10,322			0.956 (0.857–1.068)	0.974 (0.871–1.090)	
	**Cheese, times/week**								
		< 1	2,818 /	27,796			Reference	Reference	3.707
		1–2	2,461 /	23,717			1.024 (0.952–1.100)	1.005 (0.935–1.081)	
		≥ 3	758 /	7,660			0.976 (0.877–1.087)	1.001 (0.898–1.115)	
**Sleep deprivation at 3 years of age (longitudinal design)**									
	**Yogurt, times/week**								
		< 1	1,038 /	12,405			Reference	Reference	**0.042**
		1–2	1,590 /	19,022			0.999 (0.901–1.108)	0.966 (0.870–1.073)	
		3–6	1,425 /	18,386			0.926 (0.833–1.030)	0.917 (0.824–1.020)	
		≥ 7	779 /	10,565			**0.881 (0.779–0.996)**	0.897 (0.791–1.016)	
	**Cheese, times/week**								
		< 1	2,263 /	28,351			Reference	Reference	2.532
		1–2	1,935 /	24,243			1.000 (0.923–1.084)	0.979 (0.904–1.062)	
		≥ 3	634 /	7,784			1.021 (0.908–1.147)	1.047 (0.930–1.177)	

Bold indicates significance

^a^ 95% CI after application of Bonferroni correction corresponding to the 98.75% (= 100–5/4) CIs before Bonferroni correction

^b^ Adjusted for maternal age, pre-pregnancy body mass index, parity, highest education level, annual household income, marital status, alcohol intake, smoking history, employed, cesarean section, gestational weeks, birth weight, infant sex, major congenital anomalies, birth season, night cry, nursery

^c^ Values were multiplied by 4 so that the significance level after Bonferroni correction remains at 5%

## Discussion

In this study, we hypothesized that, as newborns grow, they will be influenced by the foods that they eat, and using data from 65,210 infants in the JECS, we examined the association between the frequency of consumption of two fermented foods (yogurt and cheese) at age 1 year and sleep deprivation at age 1 and 3 years. The results showed that there was no association between the frequency of yogurt intake and sleep duration at age 1, and no difference was found among the groups for sleep duration at age 3, although a trend test revealed a difference. On the other hand, no association was found for the frequency of cheese intake at both age 1 and age 3.

We have already reported an association between the active maternal intake of fermented foods during pregnancy and a lower incidence of sleep deprivation at age 1 and 3, suggesting that the maternal diet has a relatively long-term effect on child sleep [23, 24]. In addition, in the present study, a trend test showed an association between the frequency of yogurt intake at age 1 and sleep duration at age 3. In other words, we cannot rule out the possibility that children’s own active consumption of fermented foods may affect their sleep or the possible influence of what they themselves eat as they grow up. Indeed, it has been reported that the active administration of probiotics diversifies the intestinal microbiota [30], that the intestinal microbiota has a circadian rhythm, and that the intestinal microbiota is necessary for the proper regulation of the circadian rhythms [31–33]. Moreover, animal studies have shown that the gut microbiota also affects the sleep-wake cycle, a basic state transition of the brain, and that abnormalities in the gut microbiota can lead to disturbances in brain functions such as memory formation, cognitive function, mental health, and circadian rhythms. Analysis of sleep-wake states by electroencephalography and electromyography has revealed that non-REM sleep during the sleep phase is decreased in mice in which the gut microbiota was removed and, conversely, that non-REM and REM sleep during the active phase are increased compared with normal mice [14].

This study analyzed a large dataset from participants who were considered to be representative of the Japanese population [26]. The strength of the study is that it was able to adjust for a large number of covariates. On the other hand, some limitations also exist and may be related to why the hypotheses of this study were not supported. First, the questionnaire used in this study may have been affected by the fact that it did not take into account the type of yogurt or cheese consumed. In particular, there are two types of cheese: natural cheese, which contains live lactic acid bacteria and enzymes, and processed cheese, in which the lactic acid bacteria are killed by heat treatment during cheese production. This raises the question of whether the cheese consumed was actually probiotic. Probiotic cow cheese causes changes in the intestinal microbiota of mice and supports them by delivering probiotic bacteria to their intestines [34], suggesting that the type of cheese a child eats may also be important. Future studies should use methods to compare probiotic preparations containing different concentrations of microorganisms. Second, the questionnaire used in this study asked about the frequency of consumption, not the amount consumed. Further work is needed to determine the exact amount of probiotics actually consumed and changes in the intestinal microbiota by direct investigation. Third, sleep duration was measured at age 1 and at age 3; sleep duration at age 2 is not known. In other words, changes in children’s sleep duration were not captured. Fourth, for yogurt and cheese, only the frequency of intake at age 1 was studied, and changes up to age 3 were not ascertained. In particular, the frequency of cheese intake at age 1 was generally lower and more deficient than that of yogurt. Further research is needed to examine the relationship between sleep and the active intake of fermented foods after the age of 1 year and over a longer period of time. Future research should improve on these limitations and long-term studies should examine the relationship between children’s diet and sleep duration.

## Conclusion

The frequency of children’s yogurt and cheese intake at age 1 was not associated with sleep duration at age 1 or 3. However, a trend test showed a significant association between the frequency of yogurt intake at age 1 and sleep duration at age 3.

## Data Availability

Data are unsuitable for public deposition due to ethical restrictions and the legal framework of Japan. It is prohibited by the Act on the Protection of Personal Information (Act No. 57 of 30 May 2003, amendment on 9 September 2015) to publicly deposit data containing personal information. Ethical Guidelines for Medical and Health Research Involving Human Subjects enforced by the Japan Ministry of Education, Culture, Sports, Science, and Technology and the Ministry of Health, Labour, and Welfare also restrict the open sharing of the epidemiological data. All inquiries about access to data should be sent to: jecs-en@nies.go.jp. The person responsible for handling inquiries sent to this e-mail address is Dr Shoji F. Nakayama, JECS Programme Office, National Institute for Environmental Studies.

## List of abbreviations

BMI: Body mass index
CI: Confidence interval
JECS: Japan Environment and Children’s Study
JPY: Japanese Yen
OR: Odds ratio
SAS: Statistical Analysis System
